# Baseline extracellular vesicle miRNA-30c and autophagic CTCs predict chemoradiotherapy resistance and outcomes in patients with lung cancer

**DOI:** 10.1186/s40364-023-00544-y

**Published:** 2023-11-15

**Authors:** Diego de Miguel-Perez, Francisco Gabriel Ortega, Rosario Guerrero Tejada, Antonio Martínez-Única, Christine B. Peterson, Alessandro Russo, Muthukumar Gunasekaran, Andres F. Cardona, Victor Amezcua, Jose Antonio Lorente, Jose Expósito Hernández, Christian Rolfo, Maria Jose Serrano

**Affiliations:** 1 Center for Thoracic Oncology, Tisch Cancer Institute, Icahn School of Medicine at Mount Sinai, One Gustave L. Levy Place, New York, NY 10029 USA; 2 Liquid Biopsy and Cancer Interception Group, GENYO, Centre for Genomics and Oncological Research, Pfizer/University of Granada/Andalusian Regional Government, PTS Granada, Avenida de la Ilustración 114, Granada, 18016 Spain; 3 Laboratory of Genetic Identification, Legal Medicine and Toxicology Department, Faculty of Medicine, University of Granada, Avenida de la Investigación 11, Granada, 18071 Spain; 4 Biomedical Research Institute IBS-Granada, Avda. de Madrid, 15, Granada, 18012 Spain; 5 Radiation Oncology Department, Virgen de las Nieves University Hospital, Avenida de las Fuerzas Armadas 2, Granada, 18014 Spain; 6 Biostatistics, The University of Texas MD Anderson Cancer Center, 1515 Holcombe Blvd, Houston, TX 77030 USA; 7 Marlene and Stewart Greenebaum Comprehensive Cancer Center, University of Maryland School of Medicine, 22 S. Greene Street, Baltimore, MD 21201 USA; 8 Departments of Surgery and Pediatrics, Ann and Robert H. Lurie Children’s Hospital of Chicago, Feinberg School of Medicine, Northwestern University, 225 E Chicago Ave, Chicago, IL 60611 USA; 9 Luis Carlos Sarmiento Angulo Cancer Treatment and Research Center (CTIC) / Foundation for Clinical and Applied Cancer Research (FICMAC) / Molecular Oncology and Biology Systems Research Group (Fox-G), Universidad El Bosque, Bogotá, Colombia; 10 Integral Oncology Division, Virgen de las Nieves University Hospital, Av. Dr. Olóriz 16, Granada, 18012 Spain

**Keywords:** Locally advanced non-small cell lung cancer, Chemoradiotherapy, Biomarkers, Extracellular vesicles, miRNAs, Circulating tumor cells, Autophagy

## Abstract

Concurrent chemoradiotherapy (cCRT) is the mainstay of treatment for patients diagnosed with locally advanced non-small cell lung cancer (NSCLC). One significant challenge in the effectiveness of this therapy is the potential development of resistance mechanisms, where autophagy up-regulation has been proposed as a key contributing factor. However, there is a lack of reliable biomarkers to predict outcomes on these patients. Interestingly, for addressing this gap, extracellular vesicles (EVs) and circulating tumor cells (CTCs) have emerged as potential sources of such biomarkers. In this study, we investigated EV-associated miRNAs and presence of autophagic CTCs in prospectively collected serial samples from 38 patients with stage III NSCLC undergoing cCRT. Our findings revealed that non-responders exhibited low levels of baseline EV miR-375, miR-200c, and miR-30c. In particular, EV miR-30c showed high predictive value with an area under the curve of 87.2%. Low EV miR-30c and the presence of autophagic-activated CTCs emerged as independent predictive biomarkers for shorter relapse-free survival and overall survival. Furthermore, in experimental models simulating the effects of chemo- and radiotherapy, the administration of miR-30c, either through direct transfection or encapsulation into human EVs, led to the inhibition of autophagy in these cells. This is the first report demonstrating that EV miR-30c inhibits tumor autophagy and its quantification, together with autophagic-activated CTCs, could be used as biomarkers for the stratification and monitoring of patients with NSCLC undergoing cCRT, and they may hold promising potential for guiding subsequent consolidation treatment with immunotherapy or other novel therapies based on autophagy inhibitors.

**Supplementary Information:**

The online version contains supplementary material available at 10.1186/s40364-023-00544-y.

**To the editor:**

Concurrent chemoradiotherapy (cCRT) has traditionally been the recommended treatment for patients with inoperable stage-III non-small cell lung cancer (NSCLC). Nevertheless, tumor progression is commonly developed, evidencing the lack of biomarkers [1]. Liquid biopsy is the minimally-invasive and repetitive analysis of biomarkers, such as extracellular vesicles (EVs) or circulating tumor cells (CTCs), that represents a complement or alternative to tissue biopsy to provide longitudinal information on these tumors. Autophagy activation has been associated to promote cancer cell resistance to radiotherapy and chemotherapy and CTCs and EV cargo may reveal tumor heterogeneity and autophagy regulation [2]. EVs can selectively package and transfer miRNAs that regulate autophagy and tumor progression [3]. To date, few studies have evaluated the role of CTC and EV miRNAs in patients undergoing cCRT [4–6] but none has analyzed autophagy levels. Here, we evaluated EV miRNAs and autophagy-activated CTCs as biomarkers in patients with locally advanced NSCLC undergoing cCRT.

This prospective study enrolled 38 patients with median follow-up of 16.4 (range:0.1–69.1) months as well as a control group of 13 healthy donors. At first response evaluation, 8 patients (21.1%) were classified as non-responders and 32 (78.9%) patients relapsed and 28 (73.7%) patients died during the ∼6 years follow-up. First, we isolated and analyzed EVs and CTCs from blood samples collected before, during, and after treatment following our standardized protocols [7], observing a high concentration of double-membrane nanoparticles with typical EV markers and absence of other intracellular markers (Fig. 1A-C). The isolation of epithelial CTCs and their phenotypical characterization revealed positive expression of the autophagy marker LC3B in 31.6% of the patients before treatment (Fig. 1D). The analysis of a specifically selected panel of 9 miRNAs involved in NSCLC treatment resistance and autophagy was performed in EVs from samples available in 26 patients and the 13 healthy volunteers, due to logistical issues. Non-responders presented lower baseline levels of EV miR-375, miR-200c, and miR-30c in comparison to responders (Fig. 1E). The miRNA target analysis of these 3 miRNAs showed total of 131 common target genes and significant enrichment in the phosphatidylinositol-mediated signaling pathway (PI3K), critical for autophagy regulation (Supp.data). miR-30c displayed the highest predictive value with an area-under-the-curve (AUC) of 87.2% (Fig. 1F) and also showed higher expression in healthy donors in comparison to patients (*p* = 0.011). Moreover, non-responders showed more autophagy-activated CTC during treatment (*p* = 0.043) (Supp.data). After analyzing the potential impact of all variables, we observed that low pre- or during-treatment levels of EV miR-30c and autophagic CTCs were associated with shorter relapse-free survival (RFS) and overall survival (OS) in these patients (Fig. 1G) (Supp.data). These results could aid filling an important clinical need for biomarkers to predict response to consolidative treatment after cCRT [8]. The strong association between autophagy and immune regulation could suggest a potential combination of these biomarkers with ctDNA dynamics to select possible responders to cCRT and to Durvalumab. Moreover, the correlation with the PI3K pathway, could suggest a use of EVs as predictive biomarkers in patients undergoing PI3K inhibitors to potentiate the effect of chemotherapy or immunotherapy [9, 10].

**Fig. 1:**
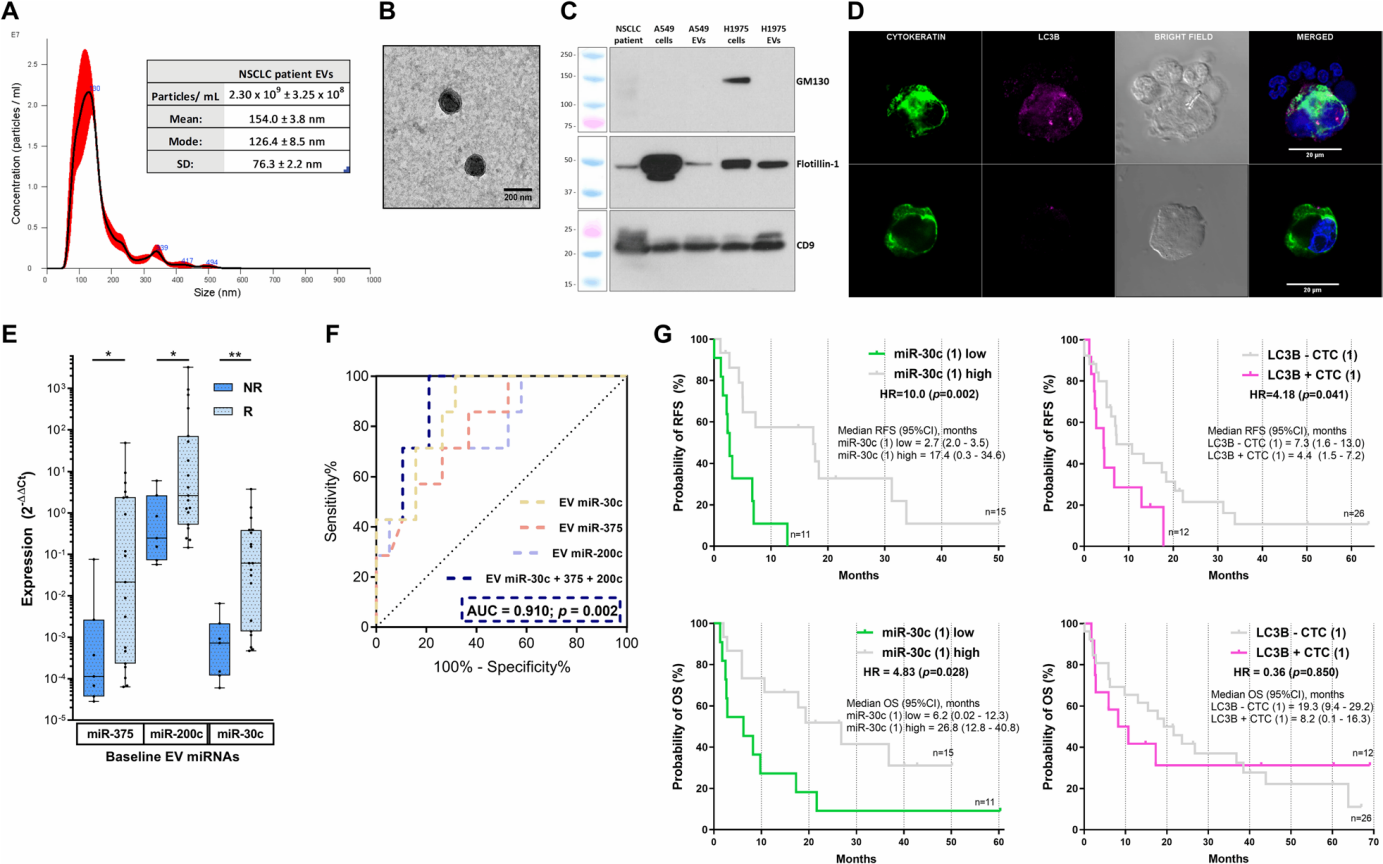
EVs and autophagic CTCs as biomarkers in patients with advanced NSCLC: **A** The Nanoparticle Tracking Analysis (NTA) of EVs from NSCLC plasma samples revealed a concentration of 2.30 × 10^9^ ± 3.25 × 10^8^ particles/ml with a diameter of mode 126.4 ± 8.5 nm. **B** The Transmission-Electron Microscopy (TEM) showed double-membrane EVs of ≈ 150 nm diameter. **C** Western Blot images from NSCLC plasma EVs and lung cancer cell lines depicted positive expression of the EV markers CD9 and Flotillin-1 while lower expression of GM-130. **D** Representative images from CTCs: The upper row shows a CTC with high LC3B expression partially surrounded by non-tumor blood cells. Lower row depicts a CTC with low LC3B. Nuclei were dyed with DAPI and images were taken with the Zeiss LSM 710 confocal/multiphoton laser scanning microscope at 63x magnification. **E** (*N* = 26) Responders showed higher baseline expression of EV miR-375, 200c, and 30c than non-responders. **F** ROC curves for the combined and individual EV miRNAs. *p < 0.05, **p < 0.01. **G** Low levels of miR-30c at baseline (1) identified patients with shorter RFS vs. high levels (*p* = 0.002). Patients with baseline autophagic-activated CTCs showed shorter RFS than those with no presence (*p* = 0.041). Low levels of baseline miR-30c also identified patients with worse OS (*p* = 0.028). Autophagic-activated CTCs (1) were not associated with OS

Second, we confirmed that chemoradiation caused down-regulation of miR-30c during autophagy up-regulation in vitro (Supp.data). This concurs with previously observed low miR-30c expression in chemotherapy-resistant lung cancer cells [11]. To evaluate the specific role of miR-30c, cells were transfected with the miRNA mimic or the inhibitor. Mimic administration resulted in reduction in cell viability and autophagy markers LC3B or Beclin-1 in comparison to the inhibitor (Fig. 2A-B & Supp.data). Increased levels of LC3B were also observed in NH_4_Cl-induced autophagy (Supp.data). Then, we also confirmed the role of EV packaged miR-30c, as cells that were incubated with miR-30c-loaded EVs showed a reduction in autophagy markers after treatment in comparison to negative controls (Fig. 2C). This supports previous studies were overexpression of miR-30c inhibited autophagy [12].

**Fig. 2:**
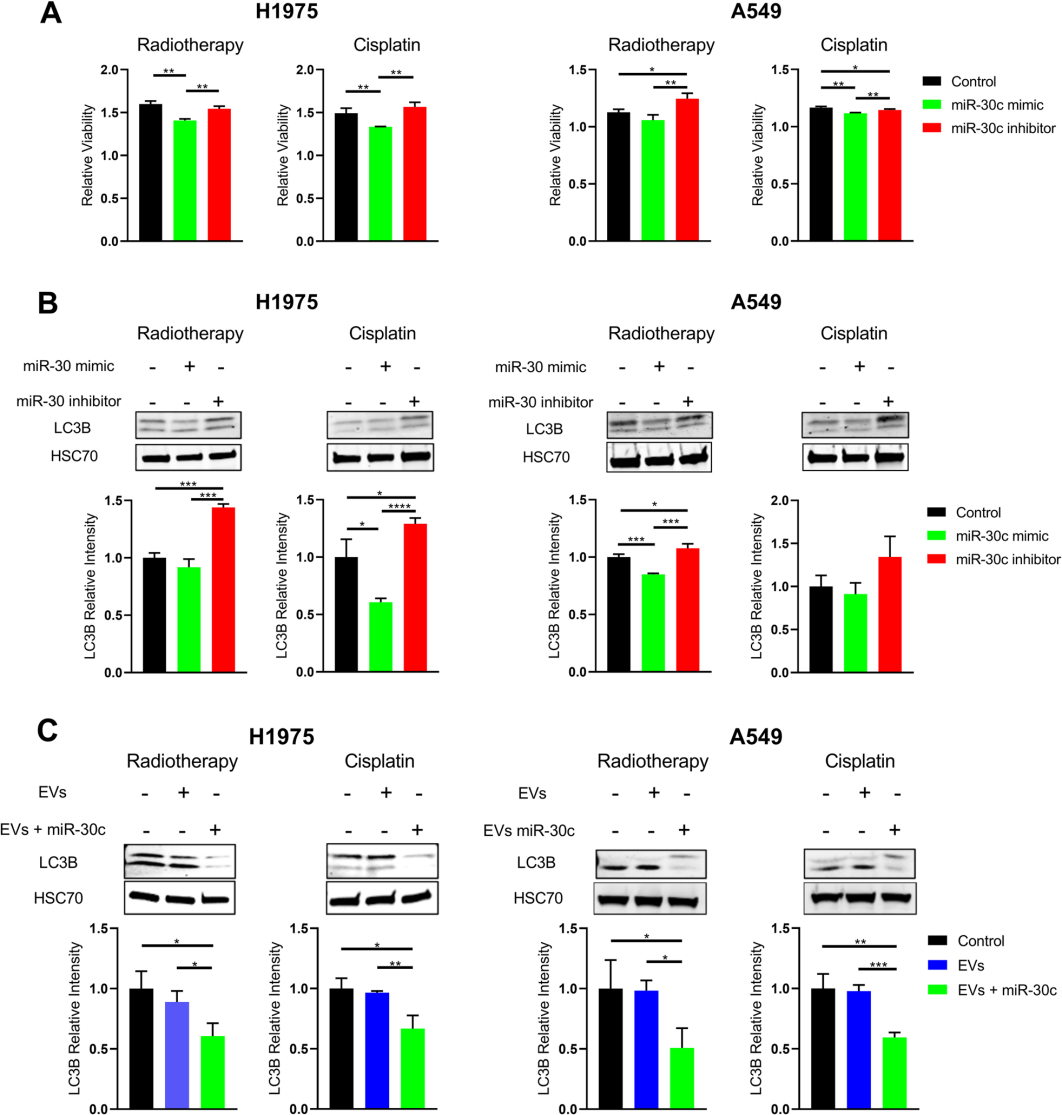
Effects of miR-30c and EV miR-30c on viability and autophagy in NSCLC cells under radiotherapy and cisplatin treatment. **A** Relative viability of H1975 and A549 cells after treatment with radiotherapy or cisplatin in controls, cells treated with miR-30c mimic, and cells with miR-30c inhibitor and **B** their representative WB images including LC3B as biomarker for autophagy quantification and HSC70 as loading control. **C** Representative WB images of cells treated with plasmatic EVs loaded/ not loaded with miR-30c mimic from independents experiments including LC3B as biomarker for autophagy quantification and HSC70 as loading control. Graphs represent media of independent experiments and standard deviation. *p < 0.05, **p < 0.01, ***p < 0.001

To sum up, we demonstrated for the first time that EV miR-30c plays a role in chemoradiation resistance and autophagy regulation, was a predictive biomarker for treatment response to cCRT, and identified patients with shorter outcomes in combination with autophagic CTCs. These results suggest that EV miR-30c and autophagic CTCs might play a significant role in the metastasis and survival of these patients and could be used as biomarkers for patient stratification to cCRT and potentially other combinatorial strategies with durvalumab or autophagy inhibitors. Nevertheless, we recognize the limitations of this preliminary study that hinders the clinical interpretation and application of these results, including the relatively small number of patients and the need to validate these results in a large multicenter study including patients receiving consolidative treatment after cCRT.

### Supplementary Information

**Additional file 1.**

## Data Availability

The datasets generated and/or analyzed during the current study are available from the corresponding authors on reasonable request.

## Abbreviations

AUC: Area-under-the-curve
cCRT: Concurrent chemoradiotherapy
CTCs: Circulating tumor cells
EVs: Extracellular vesicles
NSCLC: Non-small cell lung cancer
PI3K: Phosphatidylinositol-3-kinase
