# Supplementary material for: Baseline extracellular vesicle miRNA-30c and autophagic CTCs predict chemoradiotherapy resistance and outcomes in patients with lung cancer
Source: Biomark Res. 2023 Nov 15;11:98. doi: 10.1186/s40364-023-00544-y (PMC10652484; doi:10.1186/s40364-023-00544-y)
Supplement: Supplementary file 1 — Additional file 1. [file 40364_2023_544_MOESM1_ESM.docx]

**Supplementary data:**

**Title:**

Baseline extracellular vesicle miRNA-30c and autophagic CTCs predict chemoradiotherapy resistance and outcomes in patients with lung cancer.

**Authors:** Diego de Miguel-Perez^1,2,3^†, Francisco Gabriel Ortega^2,4^†, Rosario Guerrero Tejada^5^, Antonio Martínez-Única^5^, Christine B. Peterson^6^, Alessandro Russo^7^, Muthukumar Gunasekaran^7,8^, Andres F. Cardona^9^, Victor Amezcua^10^, Jose Antonio Lorente^2,3^, Jose Expósito Hernández^4,5^, Christian Rolfo^1^*, Maria Jose Serrano^2,4,10^*.

**Methods:**

*Study design and patients:*

This prospective longitudinal cohort study enrolled patients with unresectable locally advanced (IIIA-IIIB) NSCLC at the Virgen de las Nieves University Hospital (Granada, Spain) between September 2014 and March 2019 with last follow-up November 2020 (Supplementary Figure 1). Inclusion criteria considered individuals older than 18 years old at the time of diagnosis, with inoperable or unresectable stage IIIA‐IIIB NSCLC confirmed by histology, ECOG Performance Status ≤2, and agreement to receive concomitant treatment. Patients susceptible to palliative treatment or resectable but medically inoperable patients treated with radical radiotherapy were excluded. Additionally, a cohort of 13 healthy donors with no history of cancer were used as negative cohort for expression of EV miRNAs. Pathological stage was defined according to the 7^th^ edition of the American Joint Committee on Cancer TNM manual ^1^, the available version at the start of the study, and indication for cCRT treatment was evaluated by the Multidisciplinary Thoracic Tumor Board. Patients received standard radiation treatment with total 60 Gy, fractionated in 2Gy/daily with a 3D or IMRT technique, including primary tumor and mediastinal nodes. Treatment also included concomitant chemotherapy; 2 to 4 cycles of cisplatin plus vinorelbine (80 & 15 mg/m^2^) every three weeks or 3 to 6 cycles of carboplatin AUC2 plus paclitaxel 45 mg/m^2^ weekly. Treatment response was evaluated first by computer tomography (CT) scan at 6 to 8 weeks after the end of the treatment and at 3 months by positron emission tomography (PET)-CT. Follow-up included a CT scan every 6 months with alternating PET-CT. Response Evaluation Criteria in Solid Tumors (RECIST) version 1.1 was used to classify patients as responders (those showing complete response, partial response, or stable disease) versus non-responders (progressive disease) ^2^. Clinical outcomes were evaluated in terms of relapse-free survival (RFS) and overall survival (OS) (Patient characteristics are included in supplementary table 1).

*Blood samples:*

Peripheral blood samples were drawn before (1), at 3 weeks during concomitant treatment (2), and at its conclusion (3). Samples consisted in 15mL of peripheral blood collected in EDTA Vacutainer® tubes that were processed within 4 hours after extraction.

*EV isolation and characterization*

EVs were isolated and characterized following standardized protocols from our group in accordance to the latest recommendations of the International Society of Extracellular Vesicles ^3^. Briefly, 2mL of blood were centrifuged at 1,500 x g for 15 min for plasma collection while in the case of cell culture controls, media was centrifuged first at 500 x g for 5 min and subsequently at 3,000 x g for 15 min at 4°C. Supernatants were collected and centrifuged at 10,000 x g during 30 min at 4°C and then ultracentrifuged in 6 ml polyallomer ultracentrifuge tubes in a TFT 80.4 Rotor (ThermoFisher scientific, USA) at 100,000 × g for 70 min at 4 °C. EV pellets were directly resuspended into the tube with 1X phosphate buffered saline (PBS) for Nanoparticle Tracking Analysis (NTA) and Transmission-Electron Microscopy (TEM), in ice‐cold 1X Cell Lysis Buffer (Cell Signaling Technology) for protein characterization, or in the Maxwell® RSC miRNA lysis buffer from the Plasma and Serum Kit (AS1680) for miRNA evaluation.

First, EVs were diluted at 1:500 in PBS and injected into a NanoSight NS300 system equipped with an LM14 violet 405 nm laser module (Malvern Panalytical, UK) for size and concentration measurement. Second, for TEM analysis, EVs were resuspended using 50 μl of 1% paraformaldehyde/PBS solutions and kept overnight at 4°C. Then, they were mounted onto carbon‐coated electron microscopy grids and washed twice during 3 min with 1X PBS. EVs were negatively stained with 2% uranyl acetate before visualization in the LIBRA 120 PLUS Carl Zeiss SMT transmission electron microscope at the Centre for Scientific Instrumentation (University of Granada, Spain). Finally, characterization by Western Blot (WB) analysis was performed following standard protocols described below. Here, CD9 and Flotillin-1 were analyzed as EVs-specific biomarkers and GM130 as negative control.

*EV miRNA panel selection and analysis*

A panel of nine miRNAs (21, 222, 155, 218, 375, 200c, 129, 186, 30c) was designed based on described implication in proliferation, invasion, metastasis, radiotherapy or chemotherapy resistance, or prognosis in NSCLC with special interest into those targeting important genes in autophagy and mitosis regulation such as *BECN1, MAP1LC3B, CDK1, RAF1,* or *KIF11*. miRNAs were selected with the computation algorithm ENCORI, that predicts miRNA targets ^4^ (Supplementary Table 2). miR-16 was selected as endogenous control based on the previously shown effective role as a normalizer in EV miRNAs and other miRNA studies in lung cancer ^5–7^.

The expression of these miRNAs was analyzed according to our standardized methodology for evaluating patient and healthy plasma EV miRNAs which has already proven minimal contamination from potential co-isolated circulating miRNAs ^6^. In short, miRNAs were isolated with the Maxwell® RSC miRNA Plasma and Serum Kit (Promega, USA - AS1680) in a Maxwell® 16 automatic purificator instrument (Promega) following manufacturer’s recommendations. Complementary DNA was synthesized using the TaqMan™ Advanced miRNA cDNA Synthesis Kit (Applied Biosystems) and PCR reactions were performed in triplicate using TaqMan™ MicroRNA assay probes (Supplementary Table 2) and GoTaq® Probe qPCR Master Mix (Promega) in an Applied Biosystems 7900HT Fast Real-Time PCR System (Applied Biosystems) including triplicate non-tumor and non-template controls for each miRNA in each plate to homogenize results. Expression levels were expressed as 2^‐ΔΔCt^.

*miRNA target prediction and pathway analysis:*

miRNA target prediction analysis was performed using the online software DIANA tools MicroT-CDS with a high threshold (0.7) ^8^. Common targeted genes were identified and represented using Venny 2.1. Finally, the list of common genes was evaluated with DAVID v6.8 Functional annotation tool ^9^ to identify significantly enriched KEGG pathways and GO terms using Benjamini adjusted p-value (Supplementary table 3).

*Circulating tumor cell isolation and confocal characterization*

CTCs were isolated, enumerated, and phenotypically characterized using our standardized protocol ^7,10^. Briefly, 13mL of blood were processed by density gradient centrifugation with Histopaque-1119 (Sigma-Aldrich) to enrich the mononuclear cell fraction that was later incubated with the Carcinoma Cell Enrichment and Detection Kit with MACS Technology (Miltenyi Biotec), which isolate CTCs based on the multi‐cytokeratin specific antibody (clone = CK3‐11D5) microbeads that bind to the cytoplasmic cytokeratins (CK) 7, 8, 18, and 19. After incubation, samples were passed through columns exposed to a magnet to retain CK positive cells. After washing, the magnetic field is removed and CK positive cells are eluted and spun down onto polylysine‐coated glass slides in a cytocentrifuge (Hettich). CTCs were incubated with primary antibodies mouse FITC-anti-cytokeratin antibody (CK3-6H5) (Miltenyi Biotec, Germany) and rabbit Anti‐LC3B (ThermoFisher Scientific PA1‐16930), and then with secondary antibody Donkey anti‐Rabbit 647 (Invitrogen A‐31573). Finally, slides were mounted with VECTASHIELD mounting medium with DAPI (Vector Labs) and visualized in their full extension with a Zeiss LSM 710 confocal/multiphoton laser scanning microscope by a trained expert to enumerate and evaluate CTCs. When in doubt, each specific case is discussed by two independent experts. Autophagic-activated CTCs were defined as those with expression of LC3B based on antibody controls on cell lines (Supplementary table 4).

*Cell culture:*

The lung adenocarcinoma cell lines A549 and H1975 were acquired from the American Type Culture Collection (ATCC, USA). A549 were cultured in High Glucose DMEM (Biowest, USA) and H1975 in RPMI 1640 media (Biowest), both supplemented with 10% fetal bovine serum (Avantor, USA) and 1% penicillin/streptomycin (Biowest) in a humidified incubator with 5% CO_2_ at 37°C.

*Anticancer in vitro therapy:*

Cells were seeded in 6-well plates and radiotherapy, cisplatin, and vinorelbine treatments were administered when 90% confluency was reached. Cisplatin and vinorelbine solutions were obtained from the Virgen de las Nieves Hospital Pharmacy, Granada, Spain and radiotherapy treatment was performed in an experimental irradiator (XYLON International GmbH, Hamburg, Germany) at the Health Institute of Granada (IBS-Granada), Spain. The specific dose and concentrations were determined by first testing a dose range and identifying the one causing higher expression of autophagy biomarkers. Dose range was lower than the dose which produce 20% of cell viability reduction.

*Cell viability determination:*

Cell viability was determined by Presto Blue Assay (ThermoFisher scientific) according to manufacturer’s recommendations. Briefly, cells were incubated in 10% Presto blue/ 90% cell culture media for 1 h. Then, the supernatant was placed in a 96 wells dish to be measured at 570 and 600 nm. Viability was calculated as the ratio between 570 and 600 nm absorbances.

*WB analysis*

Relative quantification of specific biomarker’s levels was performed by WB analysis according with previously describe methods ^11^. Briefly, protein concentration was measured with the Pierce™ BCA Protein Assay Kit (ThermoFisher Scientific). Thirty μg of protein from each sample were run into Mini‐PROTEAN TGX 4–20% precast gels (Bio‐Rad, USA), transferred to nitrocellulose membranes in the X‐Cell II Blot module (Invitrogen, USA), blocked, and incubated with 1:1000 dilution of the primary antibody: mouse monoclonal anti‐CD9 (Bio Legend, USA, 312102), rabbit polyclonal anti-Flotillin-1 (Cell Signaling Technology, USA, CST-3253S), mouse monoclonal anti-GM130 (BD Biosciences, USA, 610822), rabbit polyclonal anti‐LC3B (Novus bio, USA, NB100-2220), mouse monoclonal anti-HSC70 (Santa Cruz Biotechnology, USA, sc-7298), rabbit polyclonal anti-Beclin-1 (Sigma-Aldrich, B6061), or rabbit polyclonal anti-Actin (Sigma-Aldrich, A2066) at 4°C overnight. Then, two methods were employed for band developing. First by chemiluminescence, where membranes were incubated with goat anti-rabbit HRP (CST-7074S) or horse anti-mouse HRP (CST-7076S) secondary antibodies (Cell Signaling) to be revealed using Immobilon chemiluminescent HRP substrate (Sigma-Aldrich) and read at ImageQuant LAS4000 instrument (GE Healthcare, USA). The second, by infrared Fluorescence, where membranes were incubated with goat anti-rabbit 800CW or horse anti-mouse 680RD secondary antibodies (LI-COR Biosciences, USA) to be scanned with the odyssey scanner (LI-COR).

*Cell transfection:*

miR-30c mimic (MC11060), miR-30c inhibitor (MH11060) (UGUAAACAUCCUACACUCUCAGC) or miRNA scrambled control (4464058) were purchased to ThermoFisher Scientific and transfected into H1975 and A549 cells using Lipofectamine™ RNAiMAX Invitrogen^TM^ (ThermoFisher Scientific) according to the manufacturer’s protocol. Briefly, cells were grown until 70% confluence. Then, Lipofectamine was diluted in Opti-MEM medium GIBCO^TM^ (ThermoFisher Scientific) and mimic or inhibitor sequences were diluted as well in other tube with Opti-MEM medium. The miRNA dilution was added to the Lipofectamine dilution and incubated for 5 min. Finally, Lipofectamine-miRNAs complexes were added to the cells and incubated for 12h before the cisplatin or radiotherapy treatment were administered. After 48h of treatment, cells were removed for further analysis.

*EV transfection:*

For transfection of EVs, the vesicles were isolated from pooled plasma from healthy volunteers. EVs were isolated following methods described by Vinik and Ortega et al ^12^. Briefly, 2mL of plasma were centrifuged at 200 x g for 10 min to remove cell debris and VLDL and LDL proteins floating on the top of the plasma. Then, clean plasma is fractionated by size-exclusion chromatography in SEC columns (IZON, UK). Fractions containing EVs were concentrated using 100kD Spin Filters (Pall Corporation, USA) for further transfection. These EVs were transfected using Exofect Exosome transfection kit (System BioScience, USA) according to manufacturer’s protocol. Briefly, 10μM miR-30c mimic or scramble, 2.5μL Exofect solution, and 25μg of EVs were diluted in PBS until a final volume of 200μL. After incubation for 15 min at 37°C, the mixture was immediately transferred onto ice for 30 minutes. ExoQuick-TC reagent was added and the mixture was centrifuged at 14,000 x g for 3 min to pull down the transfected EVs. The pellet was suspended in 1mL of culture media that was used to treat two wells of a 6-well culture plate. This resulted in a concentration of EVs similar to the one obtained from 1mL of plasma (25µg EV/ mL plasma). Cells were treated with 25μg EVs per 1 mL of media in further experiments.

**NH_4_Cl-induced autophagy:**

H1975 cells were cultured on cover slides inside 6-well plates as abovementioned and treated with NH_4_Cl for 24 hours. Then, slides were extracted, washed with PBS, permeabilizied in 0.1% triton for 5 minutes and fixed in 4% PFA for 15min. Cells were blocked in 5% FBS and 1% Goat serum in PBS and incubated overnight with rabbit polyclonal anti-LC3B antibody (1:100) (Novus bio, USA, NB100-2220). Cells were washed and incubated with goat anti-rabbit secondary antibody labelled with FITC (1:2000, ThermoFisher Scientific, USA). Finally, actin filaments were stained with Texas-Red phalloidin for 15 min, washed, and mounted in slides with mounting media with DAPI (Sigma Aldrich, UK). Images were obtained in the Zeiss LSM 710 confocal/multiphoton laser scanning microscope.

*Statistical analyses*

Statistical analyses and graphs were performed using IBM SPSS Statistics (version 22.0 for Windows, IBM Corp), GraphPad Prism (version 8.4 for Windows, GraphPad software), and R 3.6.3 (R Foundation for Statistical Computing). CTCs and miRNA expression were assessed as continuous (absolute number) or dichotomous variables (presence/absence and high/low, respectively). The Evaluate Cutpoints application for R was used to calculate the best miRNA expression cut‐offs at baseline levels to predict RFS and were used in subsequent time-points ^13^. Categorical variables were compared by the Fisher’s exact test. Correlations were evaluated by Spearman’s rank correlation. miRNA association with clinical characteristics or CTC presence were analyzed using the non-parametric Mann-Whitney U and Kruskal-Wallis tests. Dynamic changes of levels of miRNAs were assessed by Friedman test while the change in the presence of CTCs was measured by Cochran Q test. All data from *in vitro* experiments are expressed as mean ± standard deviation (SD) from at least three independent experiments and differences were analyzed by Student’s t test. Predictive models of treatment response were generated by logistic regression. Survival analyses were performed with the univariate Kaplan–Meier (log‐rank test) and univariate and multivariate Cox Proportional‐Hazards Regression. Variables with *p*<0.1 in the univariate analysis were included in the multivariate analysis. *p* values < 0.05 were considered statistically significant.

**Supplementary results:**

Association between the clinical characteristics and EV miRNAs or CTCs, the EV miRNA cut-offs used for outcome, and the analysis for outcome prediction are found in the following supplementary tables.Results from EV miRNA differences between cancer and healthy individuals are shown in Supplementary Figure 2. Supplementary Figure 3 shows common gene targets from the 3 miRNAs associated with response (375, 200c, and 30c) and enriched routes. Supplementary Figure 4 displays the association between on-treatment (2) expression of EV miR-30c or presence of autophagic CTCs and outcomes as well as the combined model of EV miR-30c and autophagic CTCs at baseline (1).

Most patients enrolled in this study received a combination of radiotherapy, cisplatin, and vinorelbine treatment. We evaluated the effect of each treatment in the induction of autophagy and miR-30c levels in H1975 and A549 cell lines. For that, we first determined the concentration of each drug in which, cell viability was higher than 80% at 48 h compared to the non-treated control (data not shown). Then, we stablished a lower dilution range for each treatment to evaluate autophagy and miR-30c modulation. As expected, each treatment induced autophagy in H1975 and A549 cells at 24 h (Supplementary Figure 5). Interestingly, both cell lines showed a peak in autophagy levels at a specific treatment concentration that were used in subsequent experiments: for radiotherapy 2 Gy in H1975 and 1 Gy in A549 cell lines, for cisplatin 0.6 µg/mL in H1975 and 0.4 µg/mL in A549, and for vinorelbine 0.4 µM in both cell lines. Moreover, we also observed altered miR-30c levels using these treatment concentrations (Supplementary Figure 5).

H1975 and A549 cells were transfected with miR-30c mimic, miR-30c inhibitor, or negative control and exposed to the mentioned treatments. Cell viability and LC3B levels as an autophagy biomarker were measured. miR-30c mimic and inhibitor did not affect cell viability in any of these cell lines when treated with vinorelbine (Supplementary Figure 6A). Neither miR-30c mimic nor miR-30c inhibitor altered autophagy levels in cell lines treated with vinorelbine (Supplementary Figure 6B). For this reason, experiments were performed only with radiotherapy and cisplatin treatments.Induction of autophagy in H1975 cells by increasing concentrations of NH_4_Cl revealed an overexpression of the autophagosome marker LC3B (Supplementary Figure 7).

**Supplementary figures:**


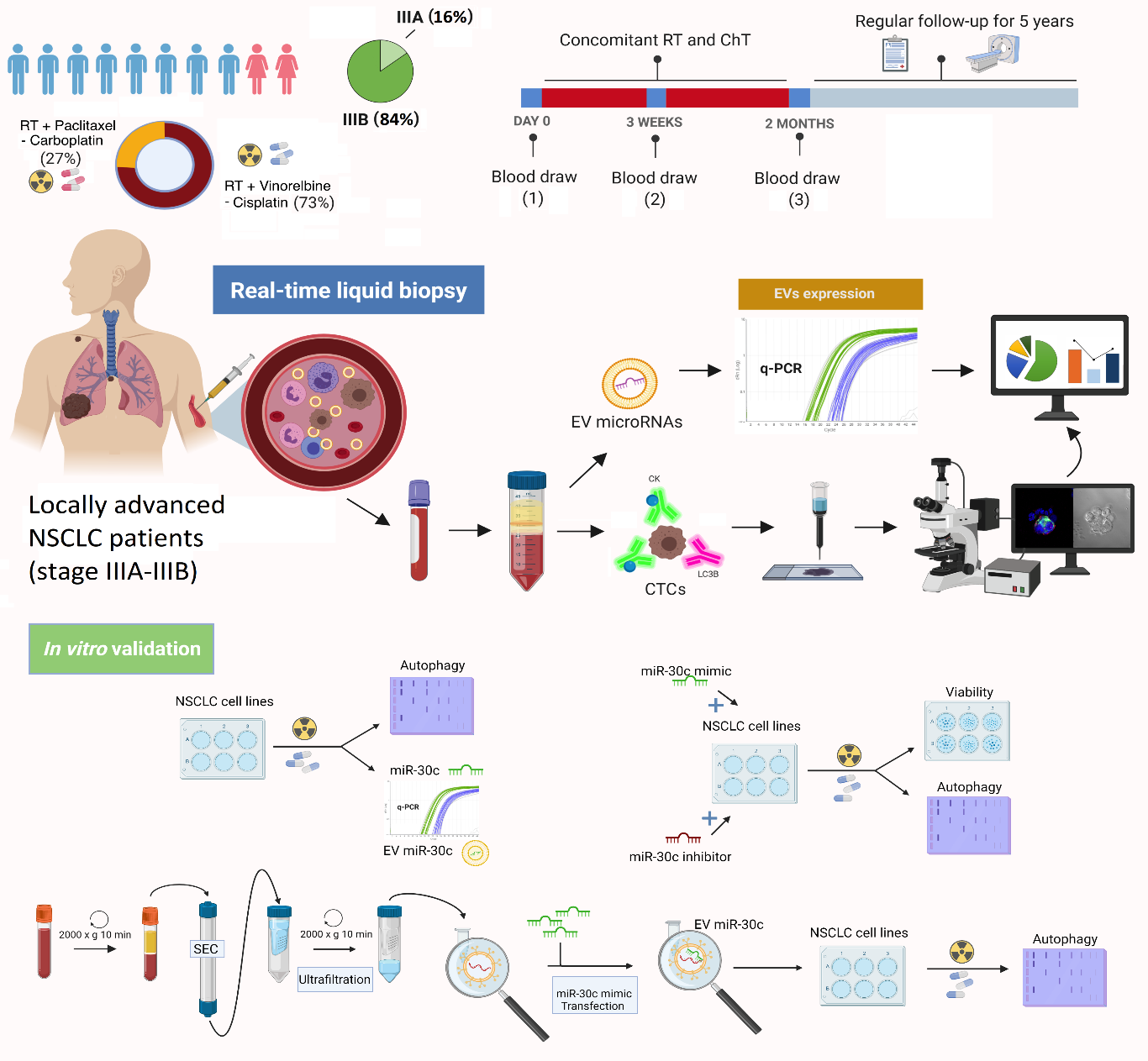


**Supplementary Figure 1:** **Study design.** Scheme of patient accrual, follow-up, and longitudinal liquid biopsy analysis of EV miRNAs and autophagic CTCs as well as *in vitro* validation (Credit: Created with Biorender).


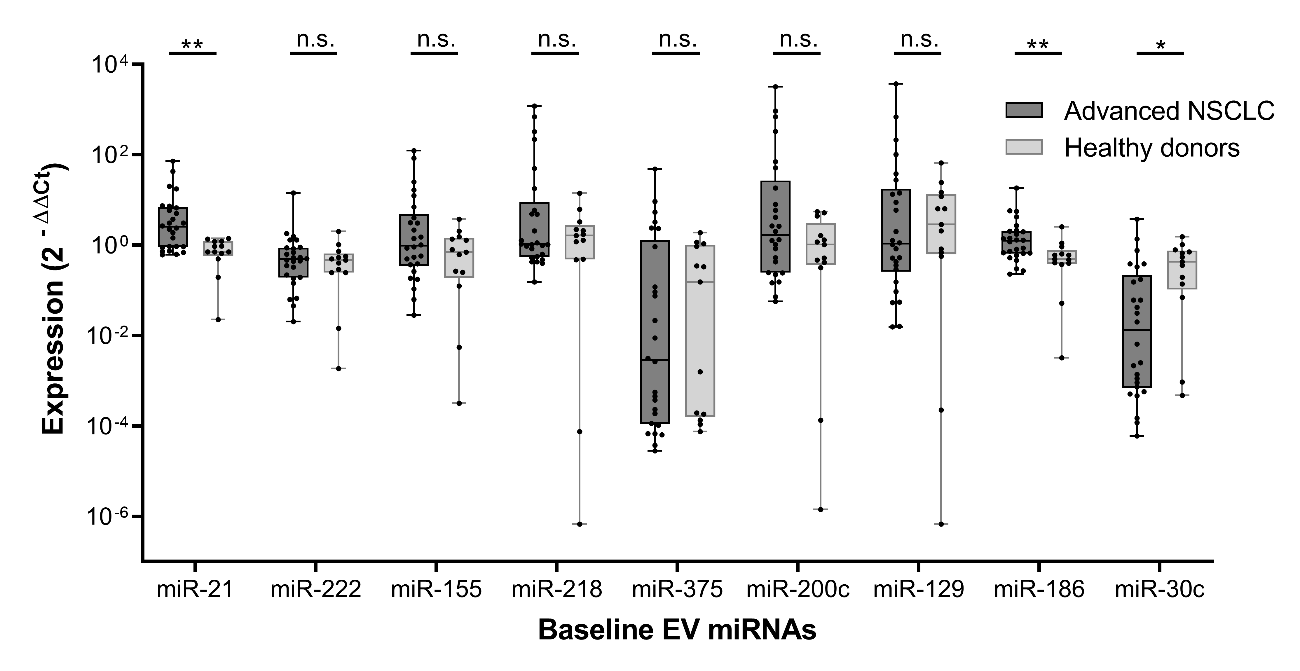


**Supplementary Figure 2: Levels of baseline EV miRNAs in patients with advanced NSCLC and healthy donors.** Patients showed higher levels of EV miR-21 (*p*=0.002) and miR-186 (*p*=0.008) while lower expression of EV miR-30c (*p*=0.011) in comparison to 13 healthy volunteers.


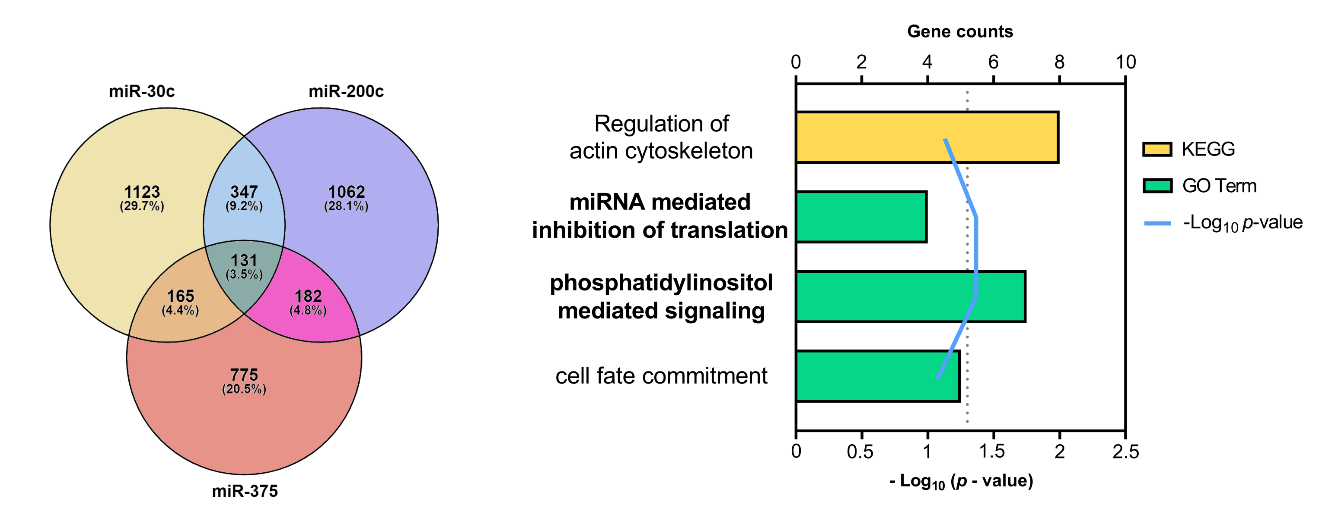


**Supplementary Figure 3:** Venn diagram showing 131 common target genes for miR-375, 200c, and 30c and KEGG pathways and GO terms enriched with common target genes from the miRNA signature, including an important regulator of autophagy such as the phosphatidylinositol-mediated signaling pathway.


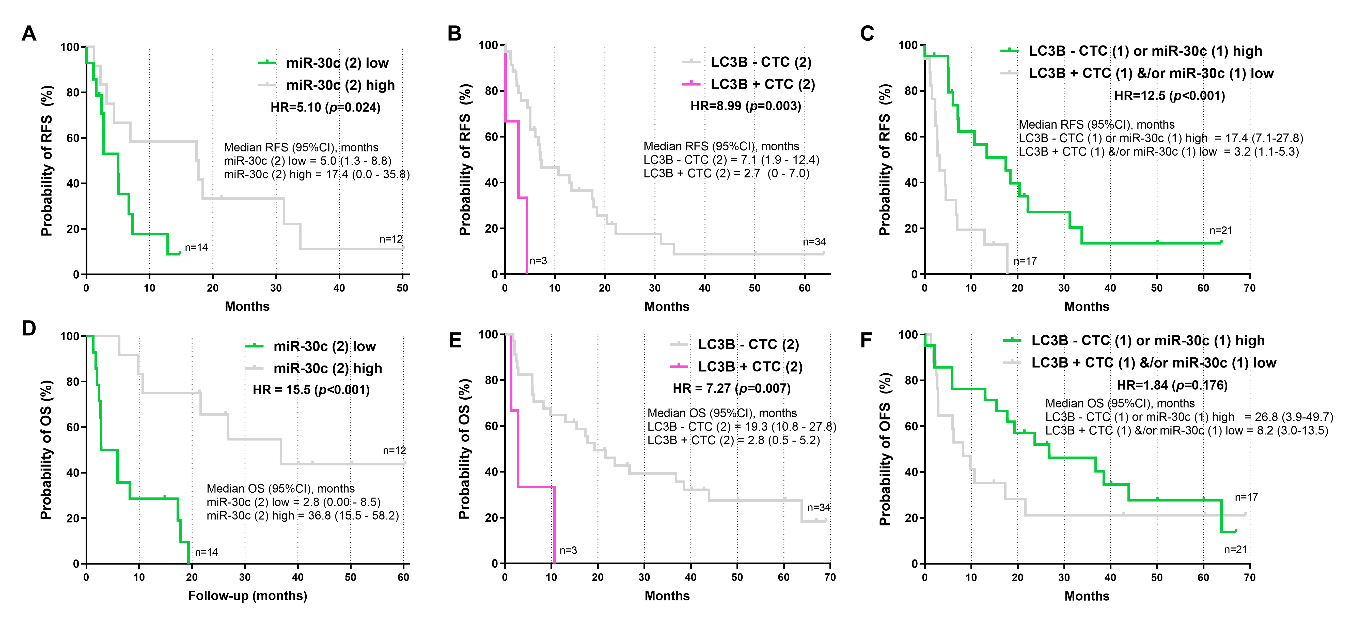


**Supplementary Figure 4: On-treatment EV miR-30c and autophagy-activated CTCs association with outcomes.** Patients with low levels of on-treatment miR-30c presented shorter RFS (*p*=0.024) (A) as well as those with presence of autophagy-activated CTCs (*p*=0.003) (B). Patients with autophagy-activated CTCs and/ or low levels of miR-30c at baseline showed shorter RFS (*p*=0.001) (C). Then, patients with shorter OS presented low levels of on-treatment miR-30c (*p*<0.001) (D) and autophagy-activated CTCs (*p*=0.007) (E). Patients with autophagy-activated CTCs and/ or low levels of miR-30c at baseline showed a trend to shorter OS (*p*=0.176) (F).


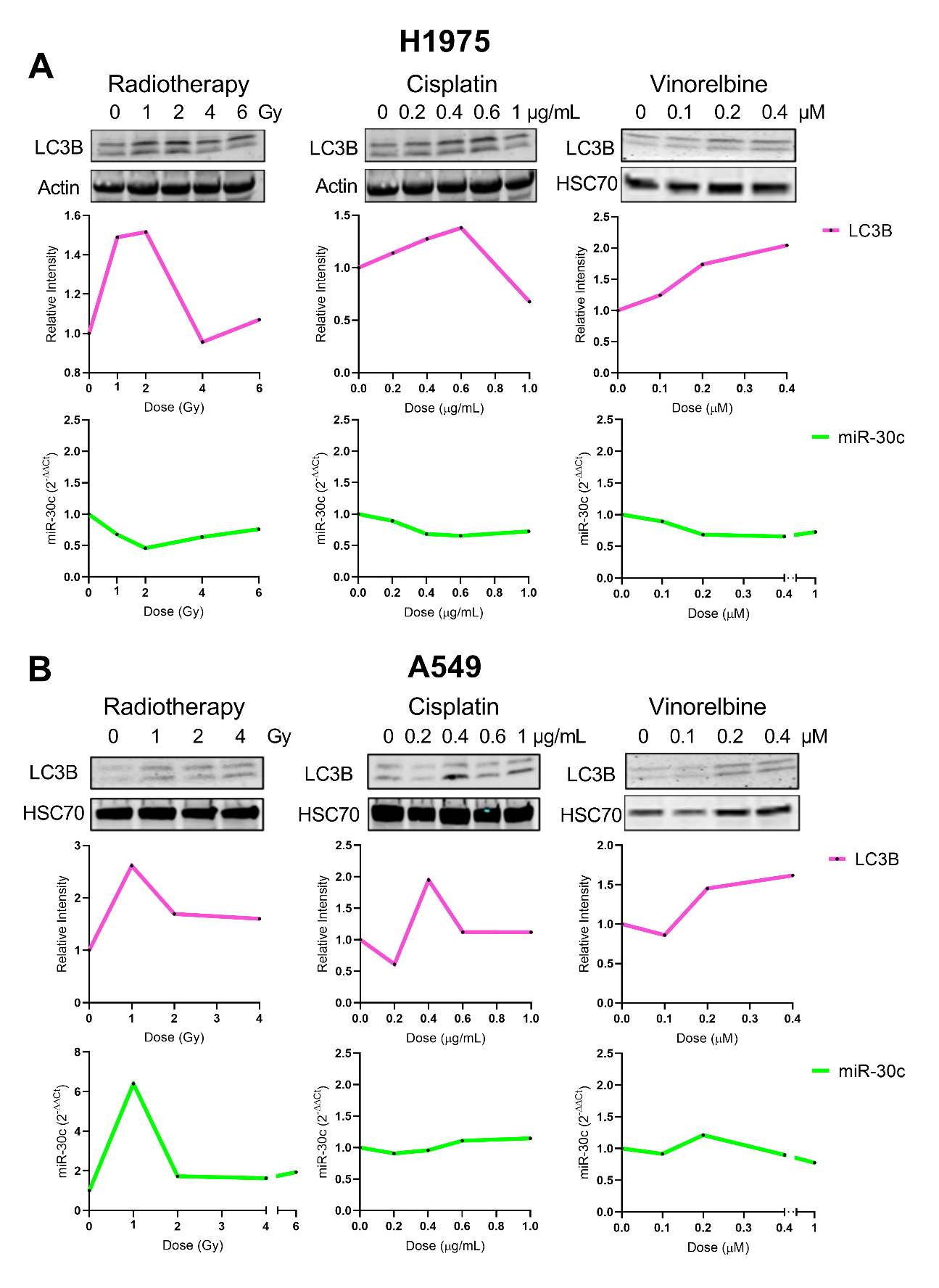


**Supplementary Figure 5: Antitumor treatments induce autophagy and affect miR-30c levels.** LC3B levels were analyzed as autophagy markers and HSC70 or Actin as loading control in WB analysis of H1975 (A) and A549 (B) cell lines after indicated treatment dose for 24 h. Graphs show the relative intensity of each band. Lower graphs show miR-30c levels after treatment.


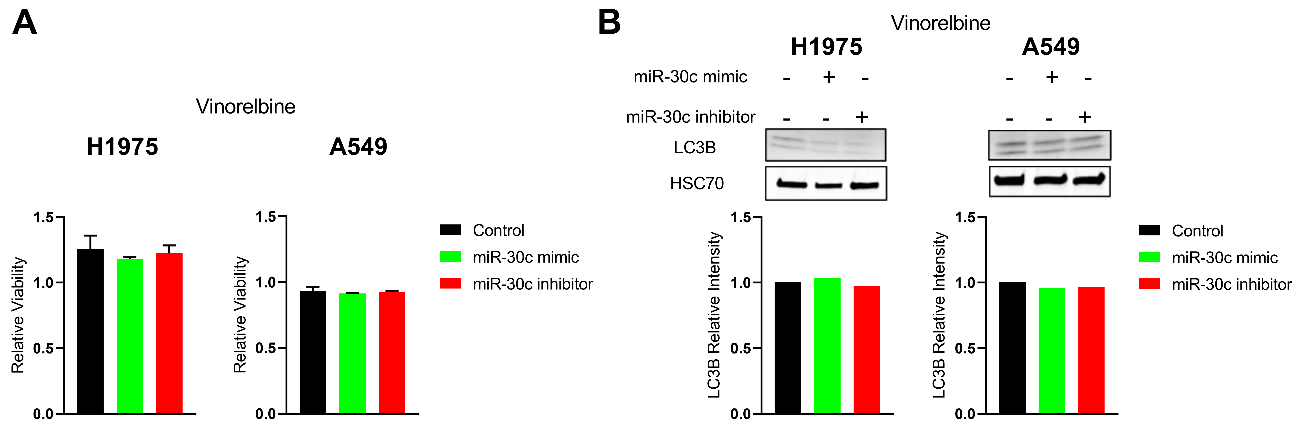


**Supplementary Figure 6:** (A) Relative viability of H1975 and A549 cells after treatment with vinorelbine in controls, cells treated with miR-30c mimic, and cells with miR-30c inhibitor. (B) WB analysis where LC3B was determined as a biomarker for autophagy quantification and HSC70 as loading control.


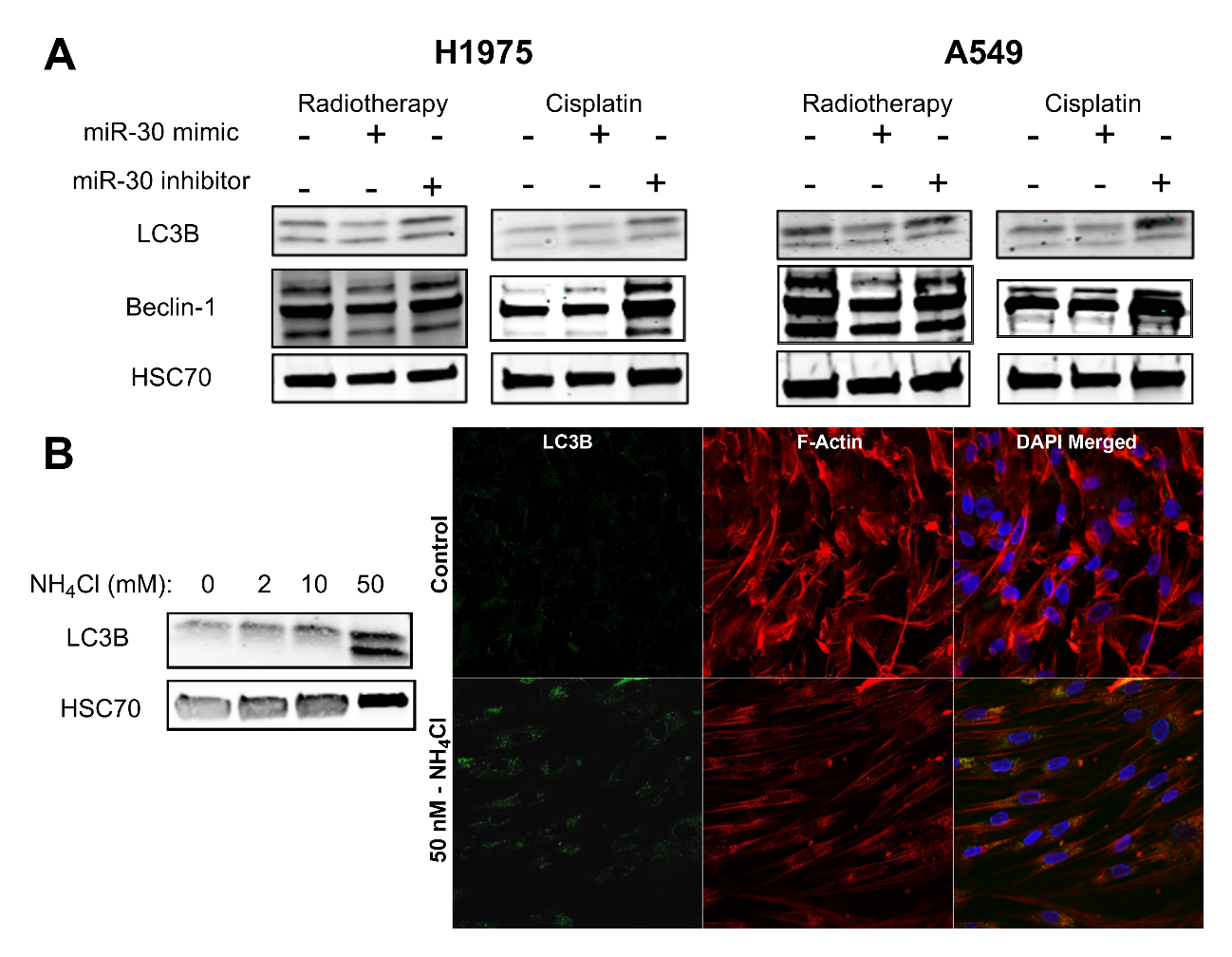


**Supplementary Figure 7:** (A) Similar to LC3B, a decrease in the expression of Beclin-1 was observed when cells were treated with the miR-30c mimic and an increase when treated with the miR-30c inhibitor during radiotherapy or cisplatin treatment. (B) Treatment with NH_4_Cl in H1975 cells induced autophagy. This was observed by the increase of LC3B expression in western-blot and immunofluorescence.

**Supplementary tables:**

**Supplementary table 1: Clinical-pathological characteristics and outcomes in patients with locally advanced NSCLC and healthy donors**

|  | | **Locally advanced NSCLC N = 38 (%)** | **Healthy donors N = 13 (%)** |
| --- | --- | --- | --- |
| **Histological subtype** | ADC | 11 (28.9%) |  |
|  | SCC | 25 (65.8%) |  |
|  | LCC | 2 (5.3%) |  |
| **Gender** | Men | 31 (81.6%) | 8 (61.5%) |
|  | Women | 7 (18.4%) | 5 (38.5%) |
| **Age (years)** | Median ± SD | 67 ± 8.00 | 63 ± 7.5 |
|  | <70 | 28 (73.7%) | 12 (92.4%) |
|  | ≥70 | 10 (26.3%) | 1 (7.6%) |
| **Smoking habits** | Never smoker | 2 (5.3%) | 6 (46.2%) |
|  | Ex-smoker | 21 (55.3%) | 1 (7.7%) |
|  | Current smoker | 15 (39.5%) | 6 (46.2%) |
| **Stage** | IIIA | 6 (15.8%) |  |
|  | IIIB | 32 (84.2%) |  |
| **N status** | N0 | 5 (13.2%) |  |
|  | N1 | 2 (5.3%) |  |
|  | N2 | 16 (42.1%) |  |
|  | N3 | 15 (39.5%) |  |
| **Chemotherapy treatment** | Vinorelbine-Cisplatin | 28 (73.7%) |  |
|  | Paclitaxel-Carboplatin | 10 (26.3%) |  |
| **EGOG at diagnosis** | 0 | 28 (73.7%) |  |
|  | 1 | 10 (26.3%) |  |
| **Treatment response** | Responder | 29 (76.3%) |  |
|  | Non-responder | 8 (21.1%) |  |
|  | NA | 1 (2.6%) |  |
| **Relapse** | No | 8 (21.1%) |  |
|  | Yes | 32 (78.9%) |  |
| **RFS (months)** | Median (range) | 6 (0-63.8) |  |
| **Exitus** | No | 10 (26.3%) |  |
|  | Yes | 28 (73.7%) |  |
| **OS (months)** | Median (range) | 16.4 (0.1-69.1) |  |

**Supplementary table 2: miRNA selection and probes:**

| **miRNA** | **Cancer association** | **Role** | **Probe sequence** |
| --- | --- | --- | --- |
| miR-21-5p | Increases tumor cell proliferation, migration, and invasion, and targets PDCD4^14^. Promotes platinum and radioresistance by targeting PTEN^15^. EV miR-21 promotes metastasis through PDCD4 inhibition^16^. | OncomiRNA | UAGCUUAUCAGACUGAUGUUGA |
| miR-222-3p | EV miR-222 enhanced proliferation, migration, invasion, gemcitabine resistance, and worse prognosis in patient^17^. Causes tumor growth suppression by S-phase arrest^18^. | OncomiRNA and tumor suppressor | AGCUACAUCUGGCUACUGGGU |
| miR-155-5p | Enhances radioresistance. Associated to good or bad prognosis depending on histological type^19^. Promotes proliferation and invasion by targeting PDCD4^20^.^.^ | OncomiRNA and tumor suppressor | UUAAUGCUAAUCGUGAUAGGGGU |
| miR-218-5p | Good prognosis by inhibiting IL-6/STAT3^21^ and EGFR^22^. Promotes platinum sensitivity by inducing apoptosis^23^. | Tumor suppressor | UUGUGCUUGAUCUAACCAUGU |
| miR-375-3p | Associated with good prognosis^24^ and reduction of brain metastasis potentially associated with VEGF and MMP-9 inhibition^25^. Inversely associated with advanced disease, and lymphatic metastasis^26^. | Tumor suppressor | UUUGUUCGUUCGGCUCGCGUGA |
| miR-200c-3p | Induces platinum sensitivity and inhibits the epithelial to mesenchymal transition (EMT) and metastasis^27^. Induces sensitivity to microtubule-targeting agents^28^. Associated to good prognosis in vinorelbine-cisplatin treated NSCLC^29^. Inhibits EMT and metastasis by targeting HMGB1^30,31^. | Tumor suppressor | UAAUACUGCCGGGUAAUGAUGGA |
| miR-129-5p | Reduced proliferation and invasion through autophagy inhibition via HMGB1^32^. Inhibits the EMT and cisplatin resistance via DLK1^33^. Associated with G1 phase arrest and apoptosis^34^. | Tumor suppressor | CUUUUUGCGGUCUGGGCUUGC |
| miR-186-5p | Reduced proliferation and metastasis^35^. Associated to paclitaxel sensitivity in vitro and in vivo and in patients^36^. Promotes tumor cell proliferation by targeting *PTEN*^37^. | OncomiRNA and tumor suppressor | CAAAGAAUUCUCCUUUUGGGCU |
| miR-30c-5p | Reduced cell invasion and EMT^38–40^. | Tumor suppressor | UGUAAACAUCCUACACUCUCAGC |
| miR-16-5p | House-keeping^5–7^ | House-keeping | UAGCAGCACGUAAAUAUUGGCG |

**Supplementary table 3: Potential signaling pathways involved in chemoradiation resistance:**

| **KEGG_PATHWAY** | | [**Genes**](https://david.ncifcrf.gov/chartReport.jsp?d-16544-p=1&d-16544-o=1&annot=30&d-16544-s=5) | [**%**](https://david.ncifcrf.gov/chartReport.jsp?d-16544-p=1&d-16544-o=1&annot=30&d-16544-s=6) | **Fold-enrichment** | ***p*-value** | ***p*-value (Benjamini)** |
| --- | --- | --- | --- | --- | --- | --- |
| Regulation of actin cytoskeleton | | 8 | 6.1 | 5.3 | 0.00057 | 0.074 |
| **GOTERM_BP_DIRECT** | | [**Genes**](https://david.ncifcrf.gov/chartReport.jsp?d-16544-p=1&d-16544-o=1&annot=30&d-16544-s=5) | [**%**](https://david.ncifcrf.gov/chartReport.jsp?d-16544-p=1&d-16544-o=1&annot=30&d-16544-s=6) | **Fold-enrichment** | ***p*-value** | ***p*-value (Benjamini)** |
| GO:0035278 | miRNA mediated inhibition of translation | 4 | 3.1 | 46.6 | 0.000073 | **0.044** |
| GO:0048015 | phosphatidylinositol-mediated signaling | 7 | 5.3 | 9.2 | 0.00011 | **0.044** |
| GO:0045165 | cell fate commitment | 5 | 3.8 | 15.2 | 0.00031 | 0.085 |

**Supplementary table 4: Circulating tumor cells in advanced NSCLC**

|  | | **Total CTCs** | | | **LC3B + CTCs** | | | |
| --- | --- | --- | --- | --- | --- | --- | --- | --- |
|  | | **Responders** | **Non-Responders** | ***p*-value** | **Responders** | **Non-Responders** | ***p*-value** |  |
| **CTC1 (N=37)*** | Mean (SD) Range | 1 (1.69) 0-7 | 2.13 (4.12) 0-12 | 0.685 | 0.79 (1.61) 0-6 | 0.5 (0.54) 0-1 | 0.533 |  |
|  | Absence | 16 (55.2%) | 4 (50%) | 0.553 | 21 (72.4%) | 4 (50%) | 0.217 |  |
|  | Presence | 13 (44.8%) | 4 (50%) |  | 8 (27.6%) | 4 (50%) |  |  |
| **CTC2 (N=37)** | Mean (SD) Range | 0.31 (0.79) 0-4 | 9 (22.3) 0-64 | 0.079 | 0.04 (0.20) 0-1 | 7.88 (20.3) 0-58 | **0.043** |  |
|  | Absence | 24 (82.8%) | 5 (62.5%) | 0.221 | 28 (96.6%) | 6 (75%) | 0.112 |  |
|  | Presence | 5 (17.2%) | 3 (37.5%) |  | 1 (3.4%) | 2 (25%) |  |  |
| **CTC3 (N=32)** | Mean (SD) Range | 0.65 (4.16) 0-10 | 3.5 (25.9) 0-11 | 0.092 | 0.15 (0.46) 0-2 | 1.83 (20.2) 0-11 | 0.644 |  |
|  | Absence | 21 (80.8%) | 3 (50%) | 0.148 | 23 (88.5%) | 5 (83.3%) | 0.584 |  |
|  | Presence | 5 (19.2%) | 3 (50%) |  | 3 (11.5%) | 1 (16.7%) |  |  |

*One patient died before response evaluation. SD: Standard Deviation. Differences evaluated by U Mann-Whitney test.

**Supplementary table 5: Association between clinical-pathological characteristics and EV miRNA expression in locally advanced NSCLC.**

|  | **Pre cCRT treatment miRNAs (1) in locally advanced NSCLC (N=26)** | | | | | | | | |
| --- | --- | --- | --- | --- | --- | --- | --- | --- | --- |
|  | **miR-21** | **miR-222** | **miR-155** | **miR-218** | **miR-375** | **miR-200c** | **miR-129** | **miR-186** | **miR-30c** |
| **Gender** | 0.316 | 0.864 | 1.000 | 0.429 | 0.130 | 0.197 | 0.197 | 0.656 | 0.560 |
| **Age (years)** | **0.006** | **0.030** | **0.004** | **0.018** | 0.063 | 0.083 | **0.013** | **0.018** | 0.572 |
| **Smoking habits** | 0.458 | 0.974 | 0.651 | 0.575 | 0.654 | 0.679 | 0.979 | 0.861 | 0.523 |
| **Histological subtype** | 0.491 | 0.312 | 0.339 | 0.833 | 0.751 | 0.874 | 0.792 | 0.874 | 0.958 |
| **Stage** | 0.656 | 0.973 | 0.864 | 0.607 | 1.000 | 1.000 | 0.515 | 0.560 | 0.197 |
| **N status** | 0.135 | 0.284 | 0.273 | 0.211 | 0.195 | 0.144 | 0.071 | 0.161 | 0.398 |
| **Chemotherapy**  **treatment** | 0.452 | 0.660 | 0.363 | 0.087 | 0.421 | 0.660 | 0.262 | 0.452 | 0.391 |
|  | **During cCRT treatment miRNAs (2) in locally advanced NSCLC (N=26)** | | | | | | | | |
| **Gender** | 0.130 | 0.973 | **0.039** | 0.656 | 0.172 | 0.864 | 0.758 | 0.352 | 0.112 |
| **Age (years)** | 0.364 | 0.497 | 0.120 | 0.821 | 1.000 | 0.461 | 0.534 | 0.534 | 0.778 |
| **Smoking habits** | 0.601 | 0.318 | 0.079 | 0.486 | 0.198 | 0.597 | 0.059 | 0.896 | **0.029** |
| **Histological subtype** | 0.833 | 0.916 | 0.458 | 0.525 | 0.241 | 0.874 | 0.426 | 0.458 | 0.241 |
| **Stage** | 0.864 | 0.656 | 0.656 | 0.316 | 0.607 | 0.656 | 0.758 | 0.706 | 0.607 |
| **N status** | 0.295 | 0.286 | 0.507 | 0.348 | 0.808 | **0.023** | 0.269 | 0.164 | 0.061 |
| **Chemotherapy**  **treatment** | 0.182 | 0.421 | **0.009** | 0.452 | 0.660 | 0.220 | 0.698 | 0.068 | 0.816 |
|  | **After cCRT treatment miRNAs (3) in locally advanced NSCLC (N=20)** | | | | | | | | |
| **Gender** | 0.479 | 0.616 | 0.216 | 0.689 | 0.921 | 0.842 | 0.842 | 10.000 | 0.179 |
| **Age (years)** | 0.933 | 0.612 | 0.866 | 1.000 | 0.230 | 0.395 | 0.866 | 0.735 | 0.553 |
| **Smoking habits** | 0.348 | 0.408 | 0.444 | 0.417 | 0.962 | 0.962 | 0.212 | 0.545 | 0.609 |
| **Histological subtype** | 0.792 | 0.734 | 0.521 | 0.473 | 0.305 | 0.157 | **0.012** | 0.427 | 0.678 |
| **Stage** | 0.616 | 0.546 | 0.305 | 0.842 | 0.093 | 0.358 | 0.358 | 0.258 | 0.216 |
| **N status** | 0.690 | 0.945 | 0.998 | 0.746 | 0.204 | 0.915 | 0.992 | 0.966 | 0.289 |
| **Chemotherapy**  **treatment** | 0.588 | 0.877 | 0.588 | 0.275 | 0.817 | 0.588 | 0.536 | 0.588 | 0.699 |

Values represent the *p*-value from Mann-Whitney U or Kruskal-wallis test. cCRT: concurrent chemoradiotherapy

**Supplementary table 6: Association between CTCs and clinical-pathological characteristics in locally advanced NSCLC.**

|  | |  |  |  |  |  |  |  |  |  |  |  |
| --- | --- | --- | --- | --- | --- | --- | --- | --- | --- | --- | --- | --- |
|  |  | **CTC (1)** | | | | **CTC (2)** | | | | **CTC (3)** | | |
|  |  | N (%) - | N (%) + | *p* | | | N (%) - | N (%) + | *p* | N (%) - | N (%) + | *p* |
| **Gender** | Men | 18 (58.1%) | 13 (41.9%) | 0.376 | 22 (73.3%) | | | 8 (26.7%) | 0.152 | 19 (76%) | 6 (24%) | 0.577 |
|  | Women | 3 (42.9%) | 4 (57.1%) |  | 7 (100%) | | | 0 (0%) |  | 5 (71.4%) | 2 (28.6%) |  |
| **Age (years)** | <70 | 13 (46.4%) | 15 (53.6%) | 0.070 | 20 (71.4%) | | | 8 (28.6%) | 0.081 | 19 (76%) | 6 (24%) | 0.577 |
|  | ≥70 | 8 (80%) | 2 (20%) |  | 9 (100%) | | | 0 (0%) |  | 5 (71.4%) | 2 (28.6%) |  |
| **Smoking habits** | Never smoker | 2 (100%) | 0 (0%) | 0.425 | 2 (100%) | | | 0 (0%) | 0.701 | 0 (0%) | 2 (100%) | **0.038** |
|  | Ex-smoker | 11 (52.4%) | 10 (47.6%) |  | 15 (75%) | | | 5 (25%) |  | 14 (77.8%) | 4 (22.2%) |  |
|  | Current smoker | 8 (53.3%) | 7 (46.7%) |  | 12 (80%) | | | 3 (20%) |  | 10 (83.3%) | 2 (16.7%) |  |
| **Histological subtype** | Non-SCC | 7 (53.8%) | 6 (46.2%) | 0.584 | 10 (76.9%) | | | 3 (23.1%) | 0.592 | 6 (50%) | 6 (50%) | **0.018** |
|  | SCC | 14 (56%) | 11 (44%) |  | 19 (79.2%) | | | 5 (20.8%) |  | 18 (80%) | 2 (20%) |  |
| **Stage** | IIIA | 3 (50%) | 3 (50%) | 0.560 | 5 (83.3%) | | | 1 (16.7%) | 0.613 | 5 (100%) | 0 (0%) | 0.211 |
|  | IIIB | 18 (56.3%) | 14 (43.8%) |  | 24 (77.4%) | | | 7 (22.6%) |  | 19 (70.4%) | 8 (29.6%) |  |
| **N status** | N0 | 3 (60%) | 2 (40%) | 0.411 | 5 (100%) | | | 0 (0%) | 0.461 | 3 (75%) | 1 (25%) | 0.820 |
|  | N1 | 0 (0%) | 2 (100%) |  | 1 (50%) | | | 1 (50%) |  | 2 (100%) | 0 (0%) |  |
|  | N2 | 10 (62.5%) | 6 (37.5%) |  | 12 (80%) | | | 3 (20%) |  | 10 (76.9%) | 3 (23.1%) |  |
|  | N3 | 8 (53.3%) | 7 (46.7%) |  | 11 (73.3%) | | | 4 (26.7%) |  | 9 (69.2%) | 4 (30.8%) |  |
| **Chemotherapy**  **treatment** | Vinorelbine based | 16 (57.1%) | 12 (42.9%) | 0.490 | 23 (85.2%) | | | 4 (14.8%) | 0.117 | 18 (72%) | 7 (28%) | 0.423 |
|  | Paclitaxel based | 5 (50%) | 5 (50%) |  | 5 (60%) | | | 2 (40%) |  | 6 (85.7%) | 1 (14.3%) |  |

**Supplementary table 7: Association between autophagic and clinical-pathological characteristics in locally advanced NSCLC.**

|  | |  |  |  |  |  |  |  |  |  |  |  |
| --- | --- | --- | --- | --- | --- | --- | --- | --- | --- | --- | --- | --- |
|  |  | **LC3B CTC (1)** | | | | **LC3B CTC (2)** | | | | **LC3B CTC (3)** | | |
|  |  | N (%) - | N (%) + | *p* | | | N (%) - | N (%) + | *p* | N (%) - | N (%) + | *p* |
| **Gender** | Men | 21 (67.7%) | 10 (32.3%) | 0.615 | 27 (90%) | | | 3 (10%) | 0.523 | 21 (84%) | 4 (16%) | 0.352 |
|  | Women | 5 (71.4%) | 2 (28.6%) |  | 7 (100%) | | | 0 (0%) |  | 7 (100%) | 0 (0%) |  |
| **Age (years)** | <70 | 18 (64.3%) | 10 (35.7%) | 0.309 | 25 (89.3%) | | | 3 (10.7%) | 0.422 | 22 (88%) | 3 (12%) | 0.648 |
|  | ≥70 | 8 (80%) | 2 (20%) |  | 9 (100%) | | | 0 (0%) |  | 6 (71.4%) | 1 (28.6%) |  |
| **Smoking habits** | Never smoker | 2 (100%) | 0 (0%) | 0.472 | 2 (100%) | | | 0 (0%) | 0.855 | 2 (100%) | 0 (0%) | 0.169 |
|  | Ex-smoker | 13 (61.9%) | 8 (38.1%) |  | 18 (90%) | | | 2 (10%) |  | 14 (77.8%) | 4 (22.2%) |  |
|  | Current smoker | 11 (73.3%) | 4 (26.7%) |  | 14 (93.3%) | | | 1 (6.7%) |  | 12 (100%) | 0 (0%) |  |
| **Histological subtype** | Non-SCC | 10 (76.9%) | 3 (23.1%) | 0.333 | 11 (84.6%) | | | 2 (15.4%) | 0.278 | 9 (75%) | 3 (25%) | 0.136 |
|  | SCC | 16 (64%) | 9 (36%) |  | 23 (95.8%) | | | 1 (4.1%) |  | 19 (95%) | 1 (5%) |  |
| **Stage** | IIIA | 3 (50%) | 3 (50%) | 0.273 | 6 (100%) | | | 0 (0%) | 0.579 | 5 (100%) | 0 (0%) | 0.488 |
|  | IIIB | 23 (71.9%) | 9 (28.1%) |  | 28 (90.3%) | | | 3 (9.7%) |  | 23 (85.2%) | 4 (14.8%) |  |
| **N status** | N0 | 3 (60%) | 2 (40%) | 0.173 | 5 (100%) | | | 0 (0%) | 0.751 | 3 (75%) | 1 (25%) | 0.746 |
|  | N1 | 0 (0%) | 2 (100%) |  | 2 (100%) | | | 0 (0%) |  | 2 (100%) | 0 (0%) |  |
|  | N2 | 12 (75%) | 4 (25%) |  | 14 (93.3%) | | | 1 (6.7%) |  | 12 (92.3%) | 1 (7.7%) |  |
|  | N3 | 11 (73.3%) | 4 (26.7%) |  | 13 (86.7%) | | | 2 (13.3%) |  | 11 (84.6%) | 2 (15.4%) |  |
| **Chemotherapy**  **treatment** | Vinorelbine based | 20 (71.4%) | 8 (28.6%) | 0.385 | 25 (92.6%) | | | 2 (7.4%) | 0.624 | 21 (84%) | 4 (16%) | 0.352 |
|  | Paclitaxel based | 6 (60%) | 4 (40%) |  | 9 (90%) | | | 1 (10%) |  | 7 (100%) | 0 (0%) |  |

**Supplementary table 8: Univariate Cox's proportional hazard regression analysis for RFS and OS**

|  | | **Univariate RFS** | | | **Univariate OS** | | |
| --- | --- | --- | --- | --- | --- | --- | --- |
|  | | **HR** | **95% CI** | ***p*** | **HR** | **95% CI** | ***p*** |
| **Gender** | Men | 1.84 | 0.64-5.31 | 0.258 | 1.59 | 0.55-4.61 | 0.393 |
|  | Women | 1.00 |  |  | 1.00 |  |  |
| **Age (years)** | <70 | 0.61 | 0.25-1.46 | 0.264 | 0.45 | 0.20-0.99 | **0.047** |
|  | ≥70 | 1.00 |  |  | 1.00 |  |  |
| **Smoking habits** | Never smoker | 1.00 |  | 0.334 | 1.00 |  | 0.522 |
|  | Ex-smoker | 0.38 | 0.08-1.68 | 0.200 | 0.53 | 0.12-2.35 | 0.401 |
|  | Current smoker | 0.31 | 0.07-1.46 | 0.139 | 0.42 | 0.09-1.94 | 0.265 |
| **Histological subtype** | Non-SCC | 1.00 | 0.48-2.10 | 0.997 | 1.13 | 0.51-2.47 | 0.770 |
|  | SCC | 1.00 |  |  | 1.00 |  |  |
| **Stage** | IIIA | 1.00 |  |  | 1.00 |  |  |
|  | IIIB | 1.13 | 0.39-3.31 | 0.819 | 0.93 | 0.32-2.72 | 0.901 |
| **N status** | N0 | 1.00 |  | 0.350 | 1.00 |  | 0.928 |
|  | N1 | 0.66 | 0.12-3.76 | 0.638 | 0.00 | 0.00-UN | 0.984 |
|  | N2 | 0.34 | 0.10-1.16 | 0.084 | 1.01 | 0.32-3.13 | 0.991 |
|  | N3 | 0.52 | 0.16-1.67 | 0.273 | 0.77 | 0.24-2.45 | 0.661 |
| **Chemotherapy**  **treatment** | Vinorelbine based | 0.91 | 0.38-2.16 | 0.833 | 0.66 | 0.29-1.54 | 0.339 |
|  | Paclitaxel based | 1.00 |  |  | 1.00 |  |  |
| **ECOG at diagnosis** | 1 | 1.26 | 0.57-2.77 | 0.572 | 1.52 | 0.66-3.50 | **0.0327** |
|  | 0 | 1.00 |  |  |  |  |  |
| **CTC1** | Presence | 0.64 | 0.30-1.34 | 0.234 | 0.69 | 0.32-1.47 | 0.336 |
|  | Absence | 1.00 |  |  | 1.00 |  |  |
| **LC3B+ CTC1** | Presence | 2.28 | 1.01-5.15 | **0.047** | 1.08 | 0.47-2.47 | 0.850 |
|  | Absence | 1.00 |  |  | 1.00 |  |  |
| **CTC2** | Presence | 0.80 | 0.33-1.98 | 0.633 | 0.93 | 0.35-2.45 | 0.875 |
|  | Absence | 1.00 |  |  | 1.00 |  |  |
| **LC3B+ CTC2** | Presence | 5.99 | 1.58-22.84 | **0.009** | 4.86 | 1.36-17.37 | **0.015** |
|  | Absence | 1.00 |  |  | 1.00 |  |  |
| **CTC3** | Presence | 2.34 | 0.99-5.52 | 0.053 | 1.01 | 0.79-4.76 | 0.150 |
|  | Absence | 1.00 |  |  | 1.00 |  |  |
| **LC3B+ CTC3** | Presence | 1.32 | 0.45-3.88 | 0.614 | 1.19 | 0.30-3.45 | 0.984 |
|  | Absence | 1.00 |  |  | 1.00 |  |  |
| **Treatment response** | Non-responders |  |  |  | 2.41 | 0.94-6.18 | 0.067 |
|  | Responders |  |  |  | 1.00 |  |  |
| **Progression** | Yes |  |  |  | 1.02 | 0.38-2.68 | 0.976 |
|  | No |  |  |  | 1.00 |  |  |

**Supplementary table 9: Cut-off categorization and Univariate Cox’s regression RFS:**

|  | **Baseline miRNA (1)** | | | | | | | | **During treatment miRNA (2)** | | | | | | **After treatment miRNA (3)** | | | | | |
| --- | --- | --- | --- | --- | --- | --- | --- | --- | --- | --- | --- | --- | --- | --- | --- | --- | --- | --- | --- | --- |
|  | **Cutpoints application** | | | **RFS** | | **OS** | | | **RFS** | | | **OS** | | | **RFS** | | | **OS** | | |
| **miRNA (High vs low)** | **Cut-off value** | ***p*** | **HR** | | ***p*** | **HR** | ***p*** | **HR** | | ***p*** | **HR** | | ***p*** | **HR** | | ***p*** | **HR** | | ***p*** |  |
| **miR-21** | 0.7284 | 0.0997 | 3.25 | | 0.119 | NA | NA | 1.78 | | 0.578 | 1.58 | | 0.657 | 0.64 | | 0.677 | 0.75 | | 0.785 |  |
| **miR-222** | 1.315 | 0.0536 | 0.59 | | 0.262 | 0.89 | 0.802 | 0.94 | | 0.889 | 0.47 | | 0.103 | 1.64 | | 0.363 | 2.70 | | 0.127 |  |
| **miR-155** | 0.176 | 0.135 | 2.53 | | 0.147 | NA | NA | 1.31 | | 0.721 | 1.36 | | 0.688 | 0.64 | | 0.677 | 0.75 | | 0.785 |  |
| **miR-218** | 1.061 | 0.142 | 0.64 | | 0.334 | 1.28 | 0.594 | 1.61 | | 0.534 | 0.89 | | 0.874 | NA | | NA | NA | | NA |  |
| **miR-375** | 2.417 | 0.0848 | 0.34 | | **0.097** | 0.32 | 0.131 | NA | | NA | NA | | NA | 1.33 | | 0.785 | 2.63 | | 0.371 |  |
| **miR-200c** | 5.982 | 0.127 | 0.37 | | **0.057** | 0.63 | 0.346 | 0.86 | | 0.741 | 0.78 | | 0.595 | 1.17 | | 0.747 | 1.88 | | 0.253 |  |
| **miR-129** | 0.3896 | 0.263 | 1.77 | | 0.269 | 1.54 | 0.442 | 1.52 | | 0.460 | 0.73 | | 0.555 | 0.45 | | 0.146 | 0.71 | | 0.560 |  |
| **miR-186** | 4.247 | 0.0434 | 0.15 | | **0.073** | 0.72 | 0.608 | 0.80 | | 0.717 | 0.81 | | 0.731 | 1.05 | | 0.940 | 1.41 | | 0.596 |  |
| **miR-30c** | 0.002154 | 0.0089 | 0.21 | | **0.004** | 0.37 | **0.034** | 0.31 | | **0.032** | 0.11 | | **0.001** | 1.33 | | 0.654 | 1.29 | | 0.741 |  |

*p*-values in bold represent variables included in the multivariate analysis.

**Supplementary references:**

1. Edge, S. B. & Compton, C. C. The american joint committee on cancer: The 7th edition of the AJCC cancer staging manual and the future of TNM. *Ann. Surg Oncol* **17**,1471–1474 (2010).

2. Eisenhauer, E. A. *et al.* New response evaluation criteria in solid tumours: Revised RECIST guideline (version 1.1). *Eur. J. Cancer* **45**, 228–247 (2009).

3. Théry, C. *et al.* Minimal information for studies of extracellular vesicles 2018 (MISEV2018): a position statement of the International Society for Extracellular Vesicles and update of the MISEV2014 guidelines. *J. Extracell. Vesicles* **7**, 1535750 (2018).

4. Li, J.-H., Liu, S., Zhou, H., Qu, L.-H. & Yang, J.-H. starBase v2.0: decoding miRNA-ceRNA, miRNA-ncRNA and protein–RNA interaction networks from large-scale CLIP-Seq data. *Nucleic Acids Res.* **42**, D92–D97 (2014).

5. Rodríguez-Martínez, A. *et al.* Exosomal miRNA profile as complementary tool in the diagnostic and prediction of treatment response in localized breast cancer under neoadjuvant chemotherapy. *Breast Cancer Res.* **21**, 21 (2019).

6. de Miguel Pérez, D. *et al.* Extracellular vesicle-miRNAs as liquid biopsy biomarkers for disease identification and prognosis in metastatic colorectal cancer patients. *Sci. Rep.* **10**, 3974 (2020).

7. De Miguel-Pérez, D. *et al.* Post-surgery circulating tumor cells and AXL overexpression as new poor prognostic biomarkers in resected lung adenocarcinoma. *Cancers (Basel).* **11**, 1750 (2019).

8. Paraskevopoulou, M. D. *et al.* DIANA-microT web server v5.0: service integration into miRNA functional analysis workflows. *Nucleic Acids Res.* **41**, W169-73 (2013).

9. Huang, D. W., Sherman, B. T. & Lempicki, R. A. Systematic and integrative analysis of large gene lists using DAVID bioinformatics resources. *Nat. Protoc.* **4**, 44–57 (2009).

10. Delgado-Ureña, M. *et al.* Circulating tumor cells criteria (CyCAR) versus standard RECIST criteria for treatment response assessment in metastatic colorectal cancer patients. *J. Transl. Med.* **16**, 251 (2018).

11. de Miguel-Perez, D. *et al.* Extracellular vesicle PD-L1 dynamics predict durable response to immune-checkpoint inhibitors and survival in patients with non-small cell lung cancer. *J. Exp. Clin. Cancer Res.* **41**, 1–14 (2022).

12. Vinik, Y. *et al.* Proteomic analysis of circulating extracellular vesicles identifies potential markers of breast cancer progression, recurrence, and response. *Sci. Adv.* **6**, eaba5714 (2020).

13. Ogłuszka, M., Orzechowska, M., Jędroszka, D., Witas, P. & Bednarek, A. K. Evaluate Cutpoints: Adaptable continuous data distribution system for determining survival in Kaplan-Meier estimator. *Comput. Methods Programs Biomed.* **177**, 133–139 (2019).

14. Yang, Y. *et al.* Downregulation of microRNA-21 expression restrains non-small cell lung cancer cell proliferation and migration through upregulation of programmed cell death 4. *Cancer Gene Ther.* **22**, 23–29 (2015).

15. MacDonagh, L. *et al.* The emerging role of microRNAs in resistance to lung cancer treatments. *Cancer Treat. Rev.* **41**, 160–169 (2015).

16. Xu, Z. *et al.* Lung adenocarcinoma cell-derived exosomal miR-21 facilitates osteoclastogenesis. *Gene* **666**, 116–122 (2018).

17. Wei, F. *et al.* Exosomes derived from gemcitabine-resistant cells transfer malignant phenotypic traits via delivery of miRNA-222-3p. *Mol. Cancer* **16**, 132 (2017).

18. Yamashita, R. *et al.* Growth inhibitory effects of miR-221 and miR-222 in non-small cell lung cancer cells. *Cancer Med.* **4**, 551–564 (2015).

19. Donnem, T. *et al.* Prognostic Impact of MiR-155 in Non-Small Cell Lung Cancer Evaluated by in Situ Hybridization. *J. Transl. Med.* **9**, 6 (2011).

20. Liu, F. *et al.* MiR-155 inhibits proliferation and invasion by directly targeting PDCD4 in non-small cell lung cancer. *Thorac. Cancer* **8**, 613–619 (2017).

21. Yang, Y. *et al.* MicroRNA-218 functions as a tumor suppressor in lung cancer by targeting IL-6/STAT3 and negatively correlates with poor prognosis. *Mol. Cancer* **16**, 141 (2017).

22. Zhu, K. *et al.* Tumor-suppressive miR-218-5p inhibits cancer cell proliferation and migration via EGFR in non-small cell lung cancer. *Oncotarget* **7**, 28075–28085 (2016).

23. Zarogoulidis, P. *et al.* MiR-205 and miR-218 expression is associated with carboplatin chemoresistance and regulation of apoptosis via Mcl-1 and Survivin in lung cancer cells. *Cell. Signal.* **27**, 1576–1588 (2015).

24. Yan, J. W., Lin, J. S. & He, X. X. The emerging role of miR-375 in cancer. *International Journal of Cancer* **135**, 1011–1018 (2014).

25. Chen, L. juan *et al.* Down-regulated microRNA-375 expression as a predictive biomarker in non-small cell lung cancer brain metastasis and its prognostic significance. *Pathol. Res. Pract.* **213**, 882–888 (2017).

26. Li, Y., Jiang, Q., Xia, N., Yang, H. & Hu, C. Decreased expression of microRNA-375 in nonsmall cell lung cancer and its clinical significance. *J. Int. Med. Res.* **40**, 1662–1669 (2012).

27. Ceppi, P. *et al.* Loss of miR-200c expression induces an aggressive, invasive, and chemoresistant phenotype in non-small cell lung cancer. *Mol. Cancer Res.* **8**, 1207–1216 (2010).

28. Cochrane, D. R., Spoelstra, N. S., Howe, E. N., Nordeen, S. K. & Richer, J. K. MicroRNA-200c mitigates invasiveness and restores sensitivity to microtubule-targeting chemotherapeutic agents. *Mol. Cancer Ther.* **8**, 1055–1066 (2009).

29. Berghmans, T. *et al.* Identification of microRNA-based signatures for response and survival for non-small cell lung cancer treated with cisplatin-vinorelbine A ELCWP prospective study. *Lung Cancer* **82**, 340–345 (2013).

30. Liu, P. L. *et al.* MicroRNA-200c inhibits epithelial-mesenchymal transition, invasion, and migration of lung cancer by targeting HMGB1. *PLoS One* **12**, e0180844 (2017).

31. Gibbons, D. L. *et al.* Contextual extracellular cues promote tumor cell EMT and metastasis by regulating miR-200 family expression. *Genes Dev.* **23**, 2140–2151 (2009).

32. Li, G., Xie, J. & Wang, J. Tumor suppressor function of mir-129-5p in lung cancer. *Oncol. Lett.* **17**, 5777–5783 (2019).

33. Ma, Z., Cai, H., Zhang, Y., Chang, L. & Cui, Y. MiR-129-5p inhibits non-small cell lung cancer cell stemness and chemoresistance through targeting DLK1. *Biochem. Biophys. Res. Commun.* **490**, 309–316 (2017).

34. Wu, J. *et al.* miR-129 regulates cell proliferation by downregulating Cdk6 expression. *Cell Cycle* **9**, 1809–1818 (2010).

35. Wang, Z., Sha, H. H. & Li, H. J. Functions and mechanisms of miR-186 in human cancer. *Biomedicine and Pharmacotherapy* **119**,109428 (2019).

36. Ye, J. *et al.* MIR-186 regulates chemo-sensitivity to paclitaxel via targeting MAPT in non-small cell lung cancer (NSCLC). *Mol. Biosyst.* **12**, 3417–3424 (2016).

37. Feng, H., Zhang, Z., Qing, X., French, S. W. & Liu, D. miR-186-5p promotes cell growth, migration and invasion of lung adenocarcinoma by targeting PTEN. *Exp. Mol. Pathol.* **108**, 105–113 (2019).

38. Xia, Y. *et al.* Down-Regulation of MiR-30c Promotes the Invasion of Non-Small Cell Lung Cancer by Targeting MTA1. *Cell. Physiol. Biochem.* **32**, 476–485 (2013).

39. Zhong, Z., Xia, Y., Wang, P., Liu, B. & Chen, Y. Low expression of microRNA-30c promotes invasion by inducing epithelial mesenchymal transition in non-small cell lung cancer. *Mol. Med. Rep.* **10**, 2575–2579 (2014).

40. Suh, S.-S. *et al.* FHIT suppresses epithelial-mesenchymal transition (EMT) and metastasis in lung cancer through modulation of microRNAs. *PLoS Genet.* **10**, e1004652 (2014).
